# Infective Arthritis.—I

**Published:** 1911-03-18

**Authors:** W. D'Oyly Grange

**Affiliations:** President of the Harrogate Medical Society.


### Hospital Clinics.

5K

### INFECTIVE ARTHRITIS.—I.

By W. D'OYLY GRANGE, M.D. Edin., President of the Harrogate Medical Society.

(Lecture delivered at the Polyclinic.)

Gentlemen,—It is an Eastern custom that conversation should begin with abject depreciation of oneself and laudation of the person addressed. With us the custom has died out, or nearly so; but, as when the flora of a district disappears one can generally find in some out-of-the-way corner a specimen or two, so we find that the custom to a certain extent may still be found to linger in presidential addresses and lectures, and lest I should contribute to the extinction of this Oriental flower of speech let me say how much it is borne in upon me that there is little new I can teach you on the subject which I have chosen for my lecture this afternoon. But, gentlemen, it is not always the function of the lecturer in this Institution to explain new discoveries and fresh methods. It is often quite as useful to go over ground which has already been broken by others and by riddling it finer to make the soil more fit for propagating the seed of definite knowledge.

### The Pathology of Joint Inflammations.

When we believe that a certain doctrine is the doctrine of the truth we cannot preach it too often or too earnestly, and I am so certain in my own mind that the theory of the infective nature of nearly all, if not all, affections of the joints is true, that I feel bound to act upon my opinion and to advance on all occasions the ideas. So much may be done to relieve, or even effect a cure in, our patients if we act in accordance with this teaching that I feel bound to urge you to accept it. You may tell me that the theory is accepted already; and I know that in some quarters it is, but in others it has received but half-hearted acceptance, and in some it is almost scouted. If we may judge by the cases which have come under my own observation, the general practitioner has not as yet given it much credence; or if he has, he has not shown his belief by making careful search after infective causes, nor has he attempted any course of treatment based upon the possibility of an infective cause still being in operation. The lack of knowledge of the truly accurate pathology of joint inflammations and enlargements is responsible for the chaos of names applied to these cases, and the question is, Are we to wait for a more accurate knowledge before we re-catalogue these diseases? Or are we justified in trying to name them on the basis of what we already know? I think myself that we are justified in attempting to frame a skeleton classification to be filled in afterwards as our knowledge becomes more accurate and the temporary callus of theory becomes ossified into facts.

### Arthritis Deformans.

In a paper on "Arthritis Deformans," by Dr. Llewellyn Jones, of Bath, read before the Balneological Section of the Royal Society of Medicine, a very good attempt was made to deal with the terminology of chronic joint diseases, and a very good clinical picture drawn of the differentiation of the two diseases known as rheumatoid arthritis and osteo-arthritis. Now in dealing with arthritis it is impossible in many cases to draw a line between the acute and the chronic forms, and in no diseases are we more justified in forming an intermediate class and labelling it subacute.

Dr. Llewellyn Jones divides cases of so-called arthritis deformans into three varieties: (1) Rheumatoid arthritis or atrophic arthritis; (2) Osteo-arthritis, or hypertrophic arthritis, (3) Infective arthritis. In describing the first group he says: "The frequency with which the disease (rheumatoid arthritis) is associated or arises in sequence to affections of avowedly infective or toxic character warrants the presumption that the malady itself is of like origin. Now, if that is the case, and I certainly think it is, the classification breaks down."

I go further than Dr. Llewellyn Jones, for to me it appears that not only is rheumatoid arthritis of infective origin, but that the much more chronic disease of osteo-arthritis is also the result of slow infection.

### Two Forms of Arthritis.

The discussion largely turned upon the differentiation between these two forms of arthritis: on the one hand between osteo-arthritis, which is most typically seen in the slow enlargement of the terminal inter-phalangeal joints in the hands of elderly people, with the production of those bony but by no means becoming enlargements, at the side of the joints, known as Heberden's nodes; and, on the other hand, the disease known as rheumatoid arthritis, seen most typically in the young woman who, on recovery from her confinement—if it may be called recovery—is afflicted with an enlargement of her wrists and ankle-joints and the proximal inter-phalangeal joints of her fingers, most deformity being seen in the middle finger. These two forms of arthritis, which, when seen side by side, are so distinct that we feel justified in giving them separate names, are sometimes so shaded into each other that it is difficult to say which is which.

### Differentiation.

The very excellent article on these joint affections by Dr. A. E. Garrod in the last edition of Clifford Allbutt's "System of Medicine" should be consulted by all interested in the subject; and I quite agree with him that now and then in the same hand we may see well-marked examples of both, the osteo-arthritis generally, I believe, having preceded the rheumatoid arthritis; as a rule but little importance is attached to the more chronic disease which has crept insidiously into his, or perhaps I should say her, life, as this disease shows a partiality for the female sex, and it is generally the unsightliness of swelling which gives most concern. I shall not, however, detain you with any further consideration of the differentiation of these two forms of arthritis. In the article referred to, and in Dr. Llewellyn Jones' books and Dr. Bannatyne's books, you will find it well discussed. I myself look upon them both as forms of infective arthritis, the one slower than the other and probably dependent on a different micro-organism differing much the same as hares and rabbits do, one finding a habitation on the surface and the other burrowing and throwing up Heberden's nodes.

### An Attempt at Classification.

You will perhaps excuse me for giving my own ideas of classification according to my own views, in no spirit of egoism but as a help towards illustrating the opinions I have formed regarding these diseases.

| | | |
|---|---|---|
| Arthritis | acute<br>subacute<br>chronic | traumatic<br>neuropathic<br>toxic<br>infective<br>septic<br>specific |

It would appear at first as if this would divide our cases on fairly well-defined lines, but it does not; for, take our first class, a traumatic arthritis is largely due to the injury to the nerves, and the slow recovery from apparently slight injury is frequently the result of nerve injury acting reflexly through the spinal centres. There appears to be a good ground for attributing to the nerve centres very marked influence upon the nutrition of the joints and in producing or preventing arthritic changes. Whether the nervous mechanism can produce the changes in the joint without the introduction of micro-organisms in the joint is a debatable point. What, however, we must all allow is that innervation of the joint, or joints, may be so enfeebled that the joint is unable to resist an infection which would have had no power against a normal innervation. In other cases we find that the virulence of the infection is so strong that it overcomes not only a normal but in some cases the most robust innervation. But to the nervous system, apart from infection, I think too extraordinary powers have been attributed. We must acknowledge the possible influence of nerve influences travelling along the vaso-motor nerves, and in one marked way we see the effect of this in the wasting which takes place in the muscles of the more acute cases.

May we not look upon this as Nature's way of treating an infection of a joint, for the muscles which are thrown out of action are the ones concerned in moving the joint, and so rest of the joint is brought about; and I am afraid we sometimes put on a masseur to work up these muscles before the joint is ready. The fear of producing a stiff joint is a bogey which prevents us from giving the rest it requires in the early stages.

### Infective Arthritis.

I think that one of the reasons for my earliest belief in the infective nature of arthritis was that I began my medical studies in the days when the antiseptic treatment of wounds was only beginning to be accepted. I was a pupil of Lister when he was working out his theories, and it interests me to see now that the same theories are being advanced to account for arthritis, and are met by very much the same arguments as were advanced to refute Lister's theory that wound suppuration is the result of bacterial infection. In the discussion at the Bacteriological Society one may recognise the same mental attitude of men who wanted to see the germ, stained and mounted, before they would acknowledge the possibility of its existence. It is rather interesting to look forward and forecast how, through the methods of serum and vaccine treatment, there is a probability of medicine getting back some of its own from surgery, and though I do not wish to see the surgeons begging their bread, yet we shall no longer see physicians eking out a precarious existence on the crumbs which fall from the surgeon's table. We who believe in the infective causes of arthritis have been challenged on the ground that if the theory be true we should by now have been able to show excellent results from the serum treatment. But surely it is early days to expect these results, though in time to come I have no doubt that the serum treatment will be by far the most valuable cure for these conditions. I hope that the profession will take up some line with regard to who is to provide these vaccines and serums, and that we shall not drift into the state of affairs which permits them to be manufactured, advertised, and sold to or by anyone.

### Scepticism.

Some people will believe nothing which is not proved to them with geometric formulæ, and because the micro-organisms have not been stained and mounted they will not believe in the possibility of their being factors in the cause of infection. These people are very useful, however, in forcing those who are of a more speculative turn of mind to prove their position, and it is often found that the truth lies between the two. I remember very well how the disciples of Lister had to fight their way to get the germ theory accepted. Let me give you an instance of what was said, and then you will appreciate that the defence is conducted on much the same lines. In the *British Medical Journal* of the early seventies we read: "Can we not by treating parallel cases with or without carbolic-acid dressings definitely decide whether on the one hand the antiseptic method is all its enthusiasts advocate it to be, or, on the other hand, save ourselves from the opprobrium of unduly vaunting a means of treatment which will not stand the test of experience?" Again, the reporter of the *British Medical Journal* says: "The germ theory and the results said to have been obtained from its application have all along been received with considerable reserve by the profession, and not the least in London."

*(To be continued.)*

